# Regulation of viral gene expression by duck enteritis virus UL54

**DOI:** 10.1038/s41598-017-01161-0

**Published:** 2017-04-21

**Authors:** Chaoyue Liu, Anchun Cheng, Mingshu Wang, Shun Chen, Renyong Jia, Dekang Zhu, Mafeng Liu, Kunfeng Sun, Qiao Yang, Ying Wu, Xinxin Zhao, Xiaoyue Chen

**Affiliations:** 1 Avian Diseases Research Center, College of Veterinary Medicine, Sichuan Agricultural University, Wenjiang, Chengdu City, Sichuan 611130 P.R. China; 2 Key Laboratory of Animal Diseases and Human Health of Sichuan Province, Wenjiang, Chengdu City, Sichuan 611130 P.R. China; 3 Institute of Preventive Veterinary Medicine, Sichuan Agricultural University, Wenjiang, Chengdu City, Sichuan 611130 P.R. China

## Abstract

Duck enteritis virus (DEV) UL54 is a homologue of human herpes simplex virus-1 (HSV-1) ICP27, which plays essential regulatory roles during infection. Our previous studies indicated that DEV UL54 is an immediate-early protein that can shuttle between the nucleus and the cytoplasm. In the present study, we found that UL54-deleted DEV (DEV-ΔUL54) exhibits growth kinetics, a plaque size and a viral DNA copy number that are significantly different from those of its parent wild-type virus (DEV-LoxP) and the revertant (DEV-ΔUL54 (Revertant)). Relative viral mRNA levels, reflecting gene expression, the transcription phase and the translation stage, are also significantly different between DEV-ΔUL54-infected cells and DEV-LoxP/DEV-ΔUL54 (Revertant)-infected cells. However, the localization pattern of UL30 mRNA is obviously changed in DEV-ΔUL54-infected cells. These findings suggest that DEV UL54 is important for virus growth and may regulate viral gene expression during transcription, mRNA export and translation.

## Introduction

Duck enteritis virus (DEV), also known as duck plague virus (DPV), is an extensively studied alpha-herpesvirus. This virus is the causative agent of duck virus enteritis (DVE), an acute haemorrhagic disease that causes significant economic losses in waterfowl-based industry due to high mortality and low egg-laying rates. The DEV genome is a linear, double-stranded DNA that is divided into a unique long region (UL) and a unique short region (US) flanked by a short internal repeat sequence (IRS) and a short terminal repeat sequence (TRS)^1^. The sequence of the complete DEV genome and the functions of several viral genes, except UL54, have been reported, which could help in eliminating DEV completely^2–4^.

As a conserved protein^5^, herpes simplex virus-1 (HSV-1) ICP27, a homologue of UL54, is required for viral replication^6^. This protein possesses a shuttling property with nuclear and cytoplasmic activities; as a result, ICP27 is multi- functional, playing roles in both the positive and the negative regulation of expression of different target genes^7–10^. In the nucleus, ICP27 can stimulate the transcription^11–15^ of early and late viral genes, affecting pre-mRNA splicing^16–20^. During dynamic shuttling, ICP27 can promote the export of viral intronless mRNAs, which are less efficiently exported than spliced RNAs^21–27^. The most important effect of this protein’s cytoplasmic activities is stimulation of the translation of certain viral transcripts^28–30^. Additionally, ICP27 has been identified to promote genomic DNA replication, which also occurs in the nucleus^31^. These findings show that HSV-1 ICP27 is important for modulating the biogenesis of DNA and mRNA, which is necessary for virus growth.

To date, there have been few reports of the role of UL54 in other herpesviruses and even fewer reports examining UL54 in DEV. In a previous study, simplified bioinformatics analyses of DEV UL54 were first conducted to lay a theoretical basis^32^. After that, DEV UL54 was expressed as a fusion protein, and in this context, a specific anti-UL54 antibody was evaluated. The intracellular localization and levels of the DEV UL54 transcript and protein were then studied, and the results showed that DEV UL54 is a nuclear protein that is expressed as early as 0.5 h after infection, with a peak at 24 h. According to a pharmacological inhibition test, UL54 was confirmed to be an immediate-early gene based on its insensitivity to the DNA polymerase inhibitor ganciclovir (GCV) and the protein synthesis inhibitor cycloheximide (CHX). Later, the DEV UL54 protein was identified to shuttle between the nucleus and the cytoplasm, and the predicted nuclear localization sequences (NLSs) and nuclear export signal (NES)^33, 34^ were evaluated. The findings suggested that DEV UL54 also plays vital regulatory roles in a similar way to HSV-1 ICP27.

In the present study, we first identified and characterized DEV-ΔUL54 and DEV-ΔUL54 (Revertant) constructs^34^. Based on results regarding the growth curve, plaque area and viral genomic DNA copy number, we found that DEV UL54 is important for virus growth. Then the viral mRNA levels, and particularly the total RNA, nuclear RNA and ribosome-nascent chain complex (RNC)-containing RNA levels, were then analysed by real-time PCR to determine the effects of DEV UL54 on viral gene expression, transcription and translation. Furthermore, the localization of UL30 mRNA in DEV-ΔUL54, DEV-ΔUL54 (Revertant) and DEV-LoxP was examined by fluorescence *in situ* hybridization (FISH). The results showed that DEV UL54 could inhibit or enhance viral gene expression, transcription and translation and promote the export of UL30 mRNA. Our results thus help to address a gap in the field of research on DEV UL54 function.

## Results

### DEV UL54 is important for viral replication

**Figure 1:**
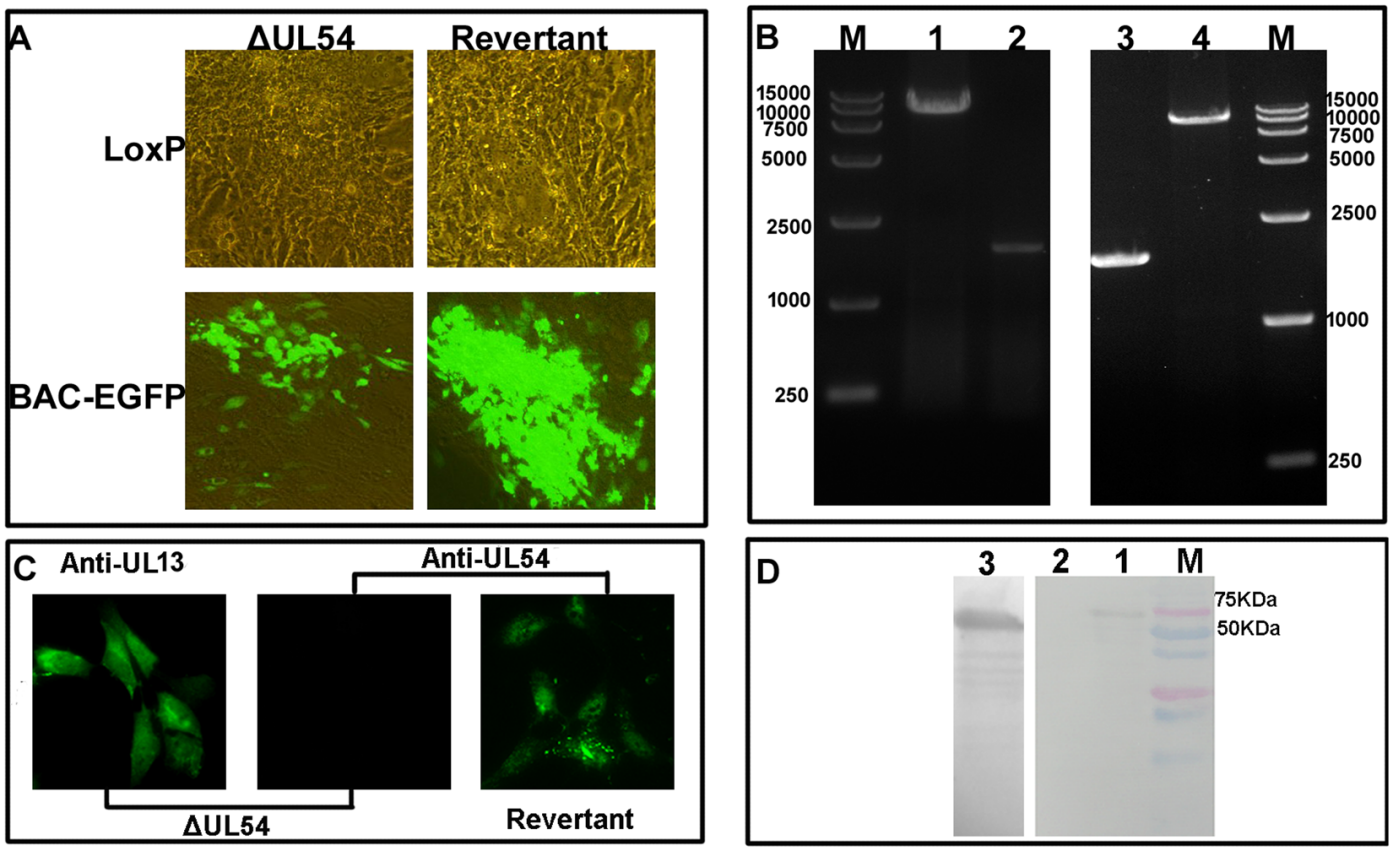
Construction of DEV-ΔUL54 and DEV-ΔUL54 (Revertant). (**A**) Construction of DEV-ΔUL54 and DEV-ΔUL54 (Revertant). The DEV CHv-BAC-ΔUL54 and DEV CHv-BAC-ΔUL54 (Revertant) were constructed with the Red recombinant system and rescued in DEFs cells. Using the Cre-LoxP system, the BAC-EGFP tag was removed. (**B**) Identification of DEV-ΔUL54 and DEV-ΔUL54 (Revertant) by PCR. M: DL15000; 1: DEV CHv-BAC-ΔUL54; 2: DEV-ΔUL54; 3: DEV-ΔUL54 (Revertant); 4: DEV CHv-BAC-ΔUL54 (Revertant). The target fragment size in DEV CHv-BAC-ΔUL54/DEV CHv-BAC-ΔUL54 (Revertant) was approximately 10000 bp, and it was nearly 1700 bp for DEV-ΔUL54/DEV-ΔUL54 (Revertant). (**C**) Identification of DEV-ΔUL54 and DEV-ΔUL54 (Revertant) by IFA. DEFs cells that were infected with DEV-ΔUL54 or DEV-ΔUL54 (Revertant) were subjected to IFA with Anti-UL54 polyclonal antibody as a primary antibody; DEV-ΔUL54 treated with Anti-UL13 polyclonal antibody as primary antibody was used as a control. (**D**) Identification of DEV-ΔUL54 and DEV-ΔUL54 (Revertant) by Western blot. Proteins of DEFs cells infected with DEV-ΔUL54 or DEV-ΔUL54 (Revertant) were subjected to Western blot with Anti-UL54 polyclonal antibody as a primary antibody. 1: DEV-ΔUL54 (Revertant); 2: DEV-ΔUL54; 3: DEV-ΔUL54 subjected with Anti-UL13 polyclonal antibody as a primary antibody.

To investigate the functional roles of DEV UL54, we first constructed DEV CHv-BAC-ΔUL54 and DEV CHv-BAC-ΔUL54 (Revertant) by genetic manipulation of DEV CHv-BAC-G^34^ (Fig. 1A, lower panels). With the help of the Cre-LoxP system, we obtained DEV-ΔUL54 and DEV-ΔUL54 (Revertant) after removing the EGFP-BAC tag (Fig. 1A, upper panels). DEV-ΔUL54 and DEV-ΔUL54 (Revertant) were identified by PCR (Fig. 1B), indirect immunofluorescence assay (IFA) (Fig. 1C) and Western blotting (Fig. 1D). The PCR results showed that the target fragments of DEV CHv-BAC-ΔUL54/DEV CHv-BAC-ΔUL54 (Revertant) and DEV-ΔUL54/DEV-ΔUL54 (Revertant) were approximately 10000 bp and 1700 bp, respectively, in size. In the IFA and Western blot analyses, no specific green fluorescence or specific band was observed for DEV-ΔUL54, which was not the case for DEV-ΔUL54 (Revertant). DEV UL13 was chosen as a control to show that the cells were successfully infected. Taken together, these results implied that the DEV UL54 deletion (DEV-ΔUL54) and revertant (DEV-ΔUL54 (Revertant)) were successfully constructed.

**Figure 2:**
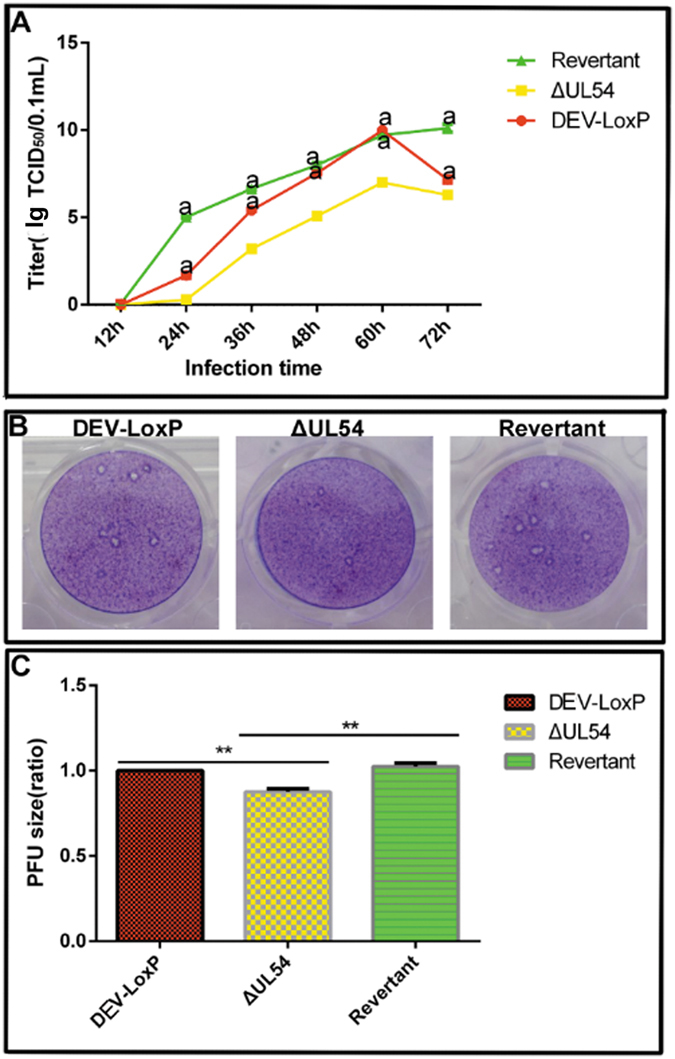
Growth curve and plaque size of the DEV-derived viruses. (**A**) Growth curve. DEFs were infected with DEV-LoxP, DEV-ΔUL54 or DEV-ΔUL54 (Revertant) at a titer of 200 TCID_50_ and harvested at different time points. The curve was generated based on the titers of different harvests by testing TCID_50_. a: p < 0.05. (**B**) Phenotypes of viral plaques. Plaques were formed on DEFs under a semi-solid medium overlay containing methylcellulose. After fixation, crystal violet staining was performed. (**C**) Analysis of the plaque area. The mean areas of plaque were analyzed using IPP 6.0. **p < 0.01.

Analyses of the growth curve and a plaque assay revealed that DEV-ΔUL54 could efficiently grow in duck embryo fibroblasts (DEFs) (Fig. 2A) while producing a smaller plaque size (Fig. 2B,C). Obvious recovery of DEV-ΔUL54 (Revertant) compared with DEV-ΔUL54 was found, suggesting that the defects resulted from the lack of the UL54 gene.

**Figure 3:**
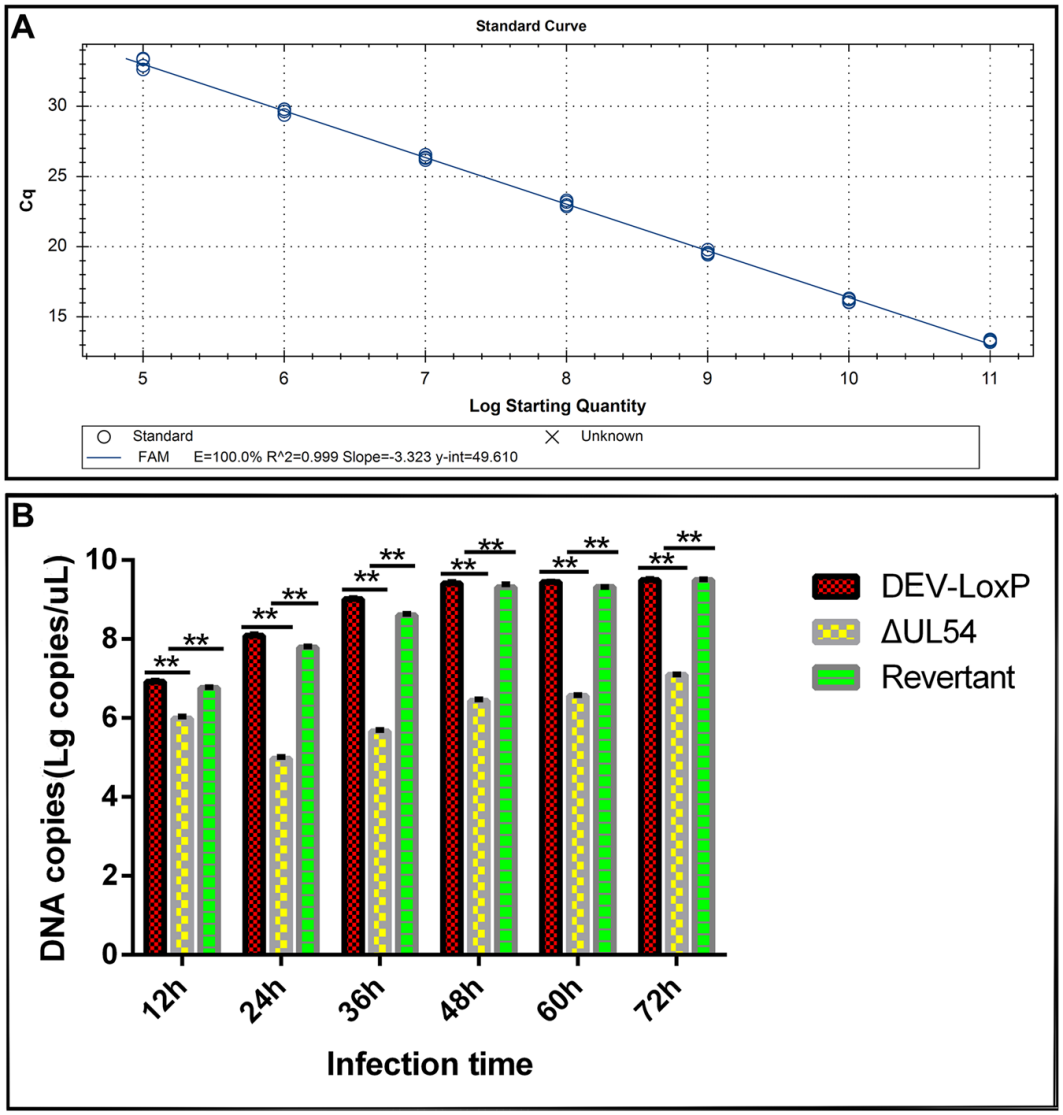
Viral DNA copy number of the DEV-derived viruses. (**A**) Construction of the standard curve. (**B**) Levels of DEV-derived genomic DNA. DEFs were infected with DEV-LoxP, DEV-ΔUL54 or DEV-ΔUL54 (Revertant) at 200 TCID_50_. At different time points, total viral DNA was purified, and quantitative PCR was performed. **p < 0.01.

Additionally, real-time PCR was performed, and the viral DNA copy number was estimated at various time points using the fitted equation of the standard curve (Fig. 3A): Y = −3.323X-49.610 (E = 100.0%, R^2 = 0.999). Compared with DEV-LoxP and DEV-ΔUL54 (Revertant), a notable decrease was observed in DEV-ΔUL54 (Fig. 3B), coinciding with the results of the growth curve and plaque assay analyses.

### DEV UL54 regulates viral gene expression during transcription and translation

**Figure 4:**
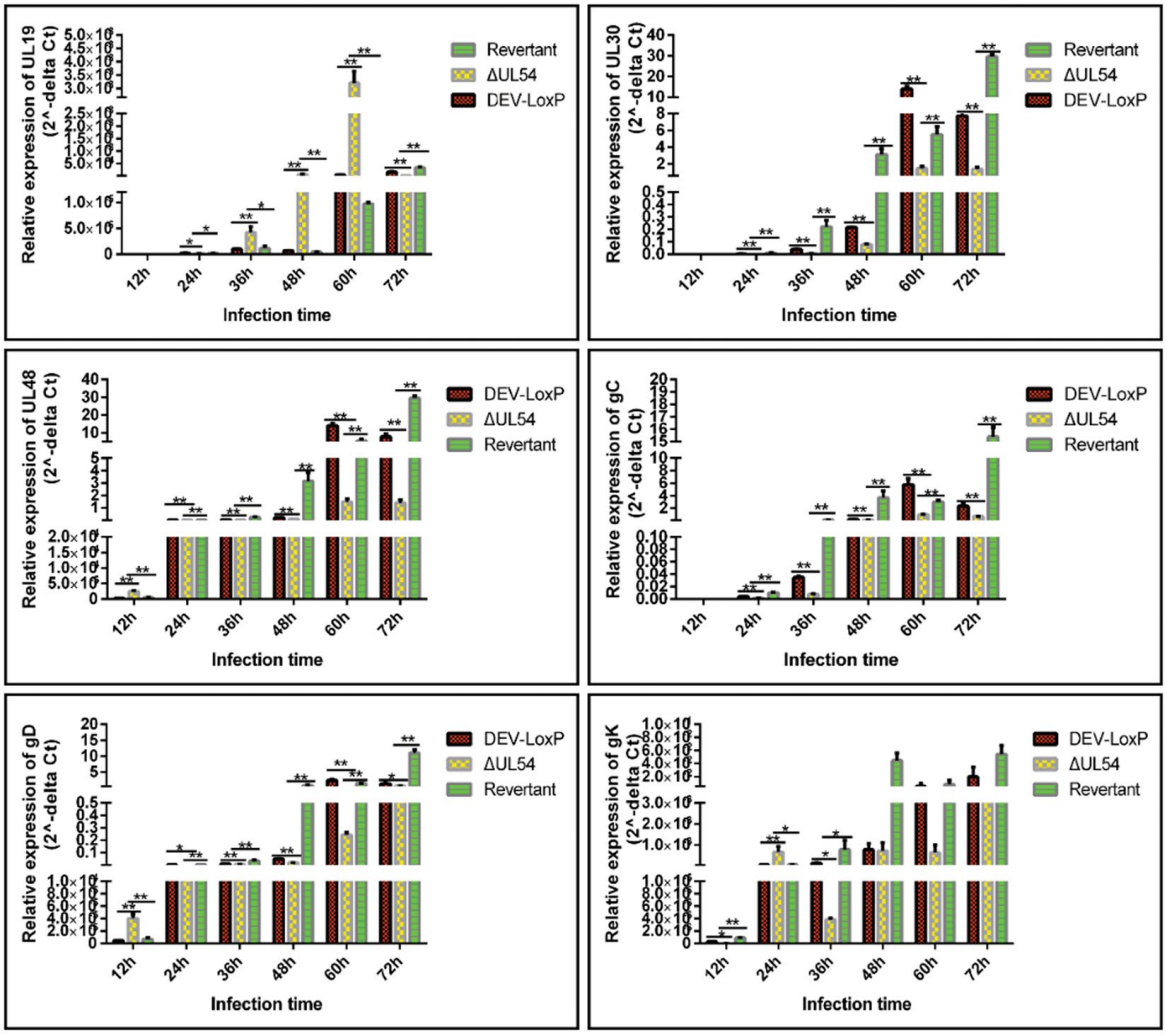
Effects of DEV UL54 on viral mRNA expression. DEFs were infected with DEV-LoxP, DEV-ΔUL54 or DEV-ΔUL54 (Revertant) at 200 TCID_50_, and RT-PCR was performed to assess the relative expression levels of UL19, UL30, UL48, gC, gD and gK at different time points. *p < 0.05; **p < 0.01.

Next, the effects of DEV UL54 on viral mRNA expression were studied. The mRNA levels of viral genes, including UL19, UL30, UL48, gC, gD, and gK were estimated by relative real-time PCR. As shown in Fig. 4, DEV UL54 continuously promoted the viral mRNA expression of UL30 and gC, and an increase was observed for UL48 and gD, except in the early stage of infection. UL19 could be both positively and negatively regulated by UL54, although the negative activity was dominant. Finally, UL54 could enhance or repress gK expression in early infection. Considering these results together, we concluded that the DEV UL54 gene could inhibit or augment viral mRNA expression.

**Figure 5:**
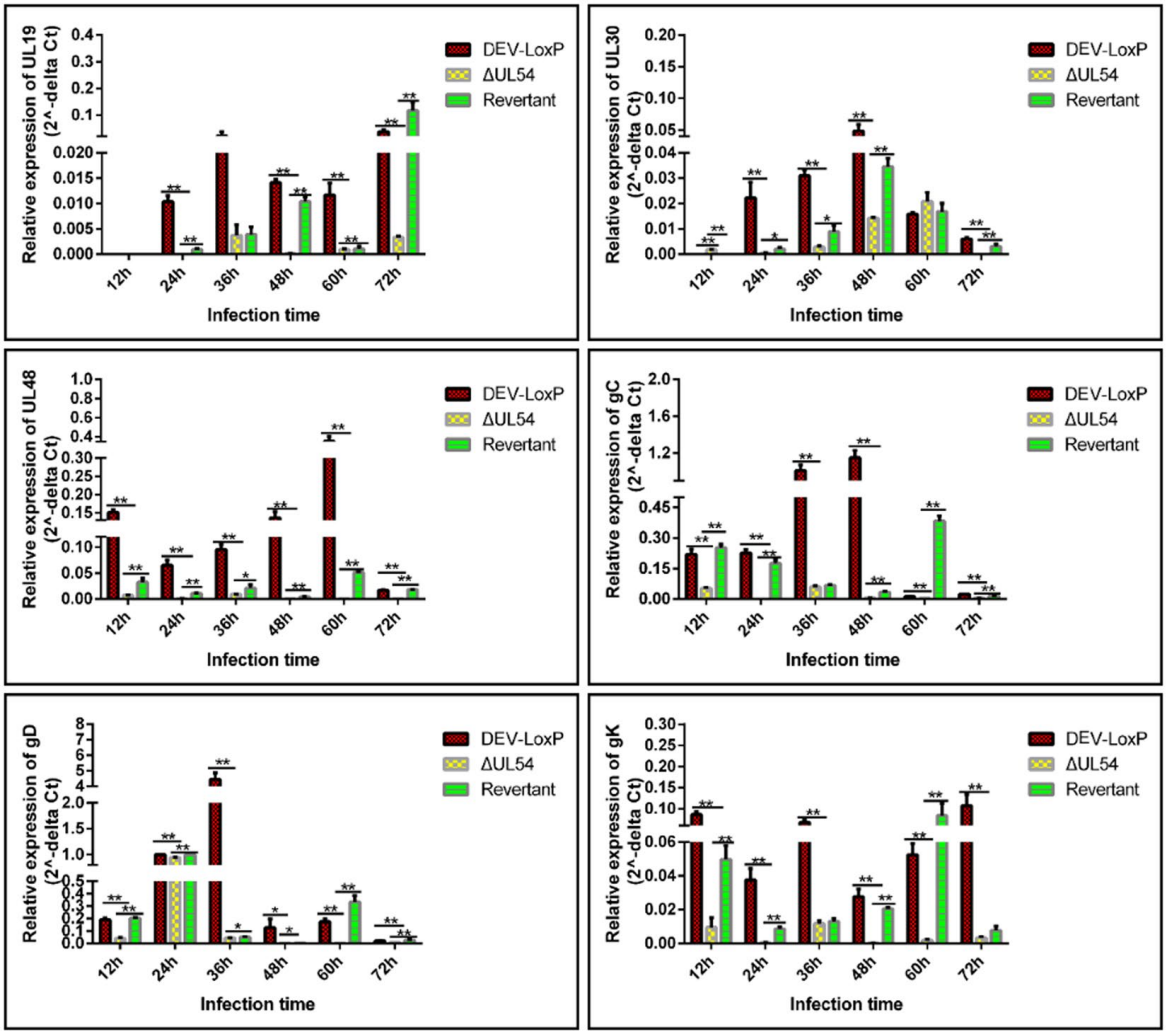
Regulation of DEV UL54 on viral gene transcription. DEFs were infected with DEV-LoxP, DEV-ΔUL54 or DEV-ΔUL54 (Revertant) at 200 TCID_50_ and the nuclei were extracted. After a nuclear run-off assay, RT-PCR was performed to assess the relative transcriptional mRNA levels of UL19, UL30, UL48, gC, gD and gK at different time points. *p < 0.05; **p < 0.01.

The transcriptional mRNA levels of UL19, UL30, UL48, gC, gD, and gK were then analysed by RT-PCR after performing a nuclear run-off assay. As shown in Fig. 5, the mRNA transcription levels for all candidate genes were significantly lower in DEV-ΔUL54 than in DPV-LoxP/DEV-ΔUL54 (Revertant), except for UL30 at 12 h. This result indicated that DEV UL54 predominantly promoted viral gene transcription during infection.

**Figure 6:**
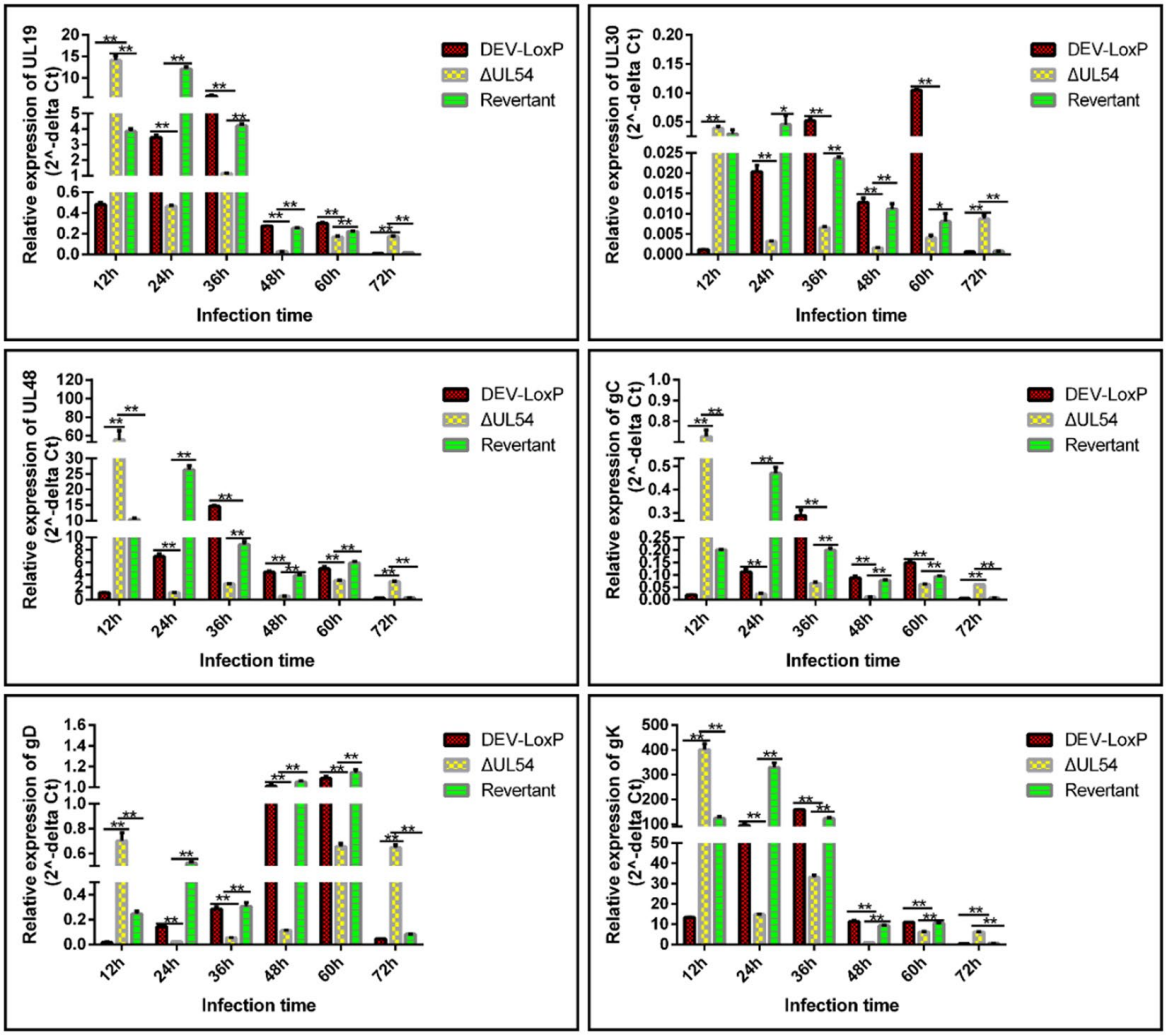
Regulation of DEV UL54 on viral gene translation. DEFs were infected with DEV-LoxP, DEV-ΔUL54 or DEV-ΔUL54 (Revertant) at 400 TCID_50_. After extracting RNC, RT-PCR was performed to assess the relative translational mRNA levels of UL19, UL30, UL48, gC, gD and gK at different time points. *p < 0.05; **p < 0.01.

As shown in Fig. 6, the mRNA translation levels for target genes were significantly lower in DEV-ΔUL54 than in DPV-LoxP/DEV-ΔUL54 (Revertant) at 12 h, whereas these levels increased from 60–72 h. This finding implied that the pattern of UL54 regulation of the translation of viral genes is as follows: inhibition in the early stage and promotion in the middle and late stages.

### DEV UL54 promotes the export of UL30 mRNA

**Figure 7:**
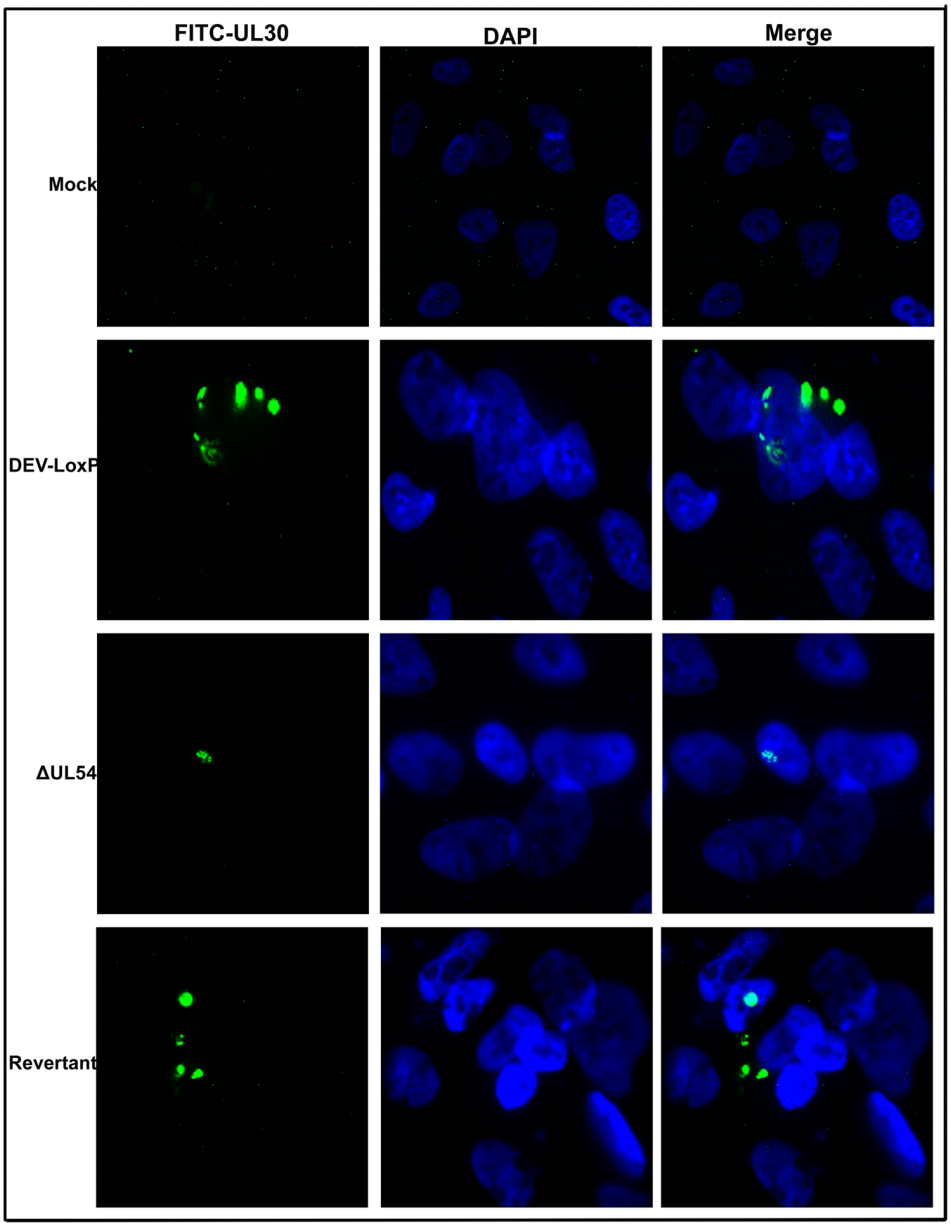
Effect of DEV UL54 on the export of UL30 mRNA. DEFs were infected with DEV-LoxP, DEV-ΔUL54 or DEV-ΔUL54 (Revertant) at 200 TCID_50_ and subjected to a FISH assay to visualize the localization of UL30 mRNA.

We investigated the export of UL30 mRNA via FISH. The results indicated that UL30 mRNA was only located in the nucleus in DEV-ΔUL54, whereas it was located in both the nucleus and the cytoplasm in wild-type and Revertant cells (Fig. 7), meaning that UL54 could prompt UL30 mRNA export.

## Discussion

The multi-functionality of HSV-1 ICP27 during infection has been well characterized^5, 21, 35^, but few reports have assessed its homologue DEV UL54. DEV UL54 is one of the immediate-early genes, which always encode proteins that are critical for regulation during infection. Therefore, we decided to study the regulatory role of DEV UL54 in viral gene expression.

Examination of the growth kinetics, plaque size and viral DNA copy number of three DEV-derived viruses generated by employing the Red recombination system showed a smaller plaque area, a lower viral titre and a lower viral DNA copy number for DEV-ΔUL54, which could be recovered. This finding indicated that the UL54 gene is important for DEV replication.

Next, the relative expression levels of UL19, UL30, UL48, gC, gD and gK, which belong to different genotypes and have different functions, were analysed by real-time PCR. The results showed that DEV UL54 could regulate viral gene expression either positively or negatively. To learn more about the effects of DEV UL54 on viral gene expression, the relative levels of mRNA transcription and translation were analysed after performing a nuclear run-off assay and RNC extraction, respectively. The results demonstrated that DEV UL54 could inhibit or augment viral gene transcription and translation. Interestingly, inhibition of UL30 by UL54 in the early stage did not cause a decrease in the total mRNA expression level, suggesting that UL54 may facilitate the export of UL30 mRNA, which was confirmed via a direct FISH assay. The shuttling property of the DEV UL54 protein may be important in this process.

The results for DEV UL54 in our study are consistent with reported findings for HSV-1 ICP27^6, 9, 10, 36^, and high conservation may be responsible for this similarity. In the present study, we analysed gene expression only on the mRNA level; we did not study protein levels due to the lack of a polyclonal antibody. Although the analysis of translation may be a proxy to a certain extent, actual protein detection would be ideal. However, an “FRT” scar remained in the recombinant virus due to application of the Red recombination system, and the effects of an “FRT” scar on viral characteristics are unclear. A new system for the construction of recombinant viruses without a scar is thus being researched.

In summary, our results demonstrate that DEV UL54 could both positively and negatively regulate viral gene expression during transcription and translation and could promote the export of UL30 mRNA. Our research opens the door to studying the function of the UL54 gene, but our study was too shallow to characterize the detailed mechanisms of expression regulation by DEV UL54. Additionally, the domains responsible for regulation of DEV UL54 should also be examined.

## Materials and Methods

### Cells and viruses

Monolayer cultures of DEFs were grown in modified Eagle’s medium (MEM) supplemented with 10% new born bovine serum (NBS) and maintained at 37 °C in a 5% CO_2_ humidified incubator. Anti-UL54 polyclonal antibody, anti-UL13 polyclonal antibody, DEV-LoxP, DEV-ΔUL54, and DEV-ΔUL54 (Revertant) were all generated in our laboratory.

### IFA and Western blotting

After incubation with DEFs for 2 h, any unadsorbed virus was discarded. The mixture of DEFs and DEV-derived virus was kept in MEM supplemented with 2% NBS and maintained at 37 °C in a 5% CO_2_ humidified incubator for 48 h.

To perform IFA, cells infected with DEV-ΔUL54 or DEV-ΔUL54 (Revertant) were sequentially treated with 4% paraformaldehyde, 0.5% Triton X-100, anti-UL54 polyclonal antibody and FITC-conjugated goat anti-rabbit IgG. Fluorescence microscopy was then applied to image the cells^37^.

Western blotting was performed as per standard protocols^38^. First, the cells infected with DEV-ΔUL54 or DEV-ΔUL54 (Revertant) were lysed, and the proteins were separated. Second, the separated proteins were transferred to polyvinylidene fluoride (PVDF) membranes. After blocking with BSA and incubation with anti-UL54 polyclonal antibody and goat anti-rabbit HRP-labelled IgG, the membranes were developed using a DAB kit (TIANGEN, PA110).

**Table 1:** Primers for real-time PCR.

Primer name	Sequence(5′-3′)	Size (bp)
β-actin(F)	CCGGGCATCGCTGACA	177
β-actin(R)	GGATTCATCATACTCCTGCTTGCT	
UL19(F)	TCTATACGAAGCACAGATGGAC	92
UL19(R)	ATGCAACAGAAAGACCCAA	
UL30(F)	GAGAAAAGCAATACGAGCCAAGA	121
UL30(R)	ACTCACTCCACAGAACCCATACAC	
UL48(F)	TGCCTGTCATACGTTGTG	134
UL48(R)	AATACGCTTCCATCTTGC	
gC(F)	GAATAAACAACCGGAACTGCT	132
gC(R)	GTCTTTGATCGGTTCGCTTC	
gD(F)	TGGTTCAAAGTCGGAGTGGG	168
gD(R)	AATGACCAGTCCGAGTTCGT	
gK(F)	CGTATTGTATCTGCGGCT	164
gK(R)	CGAGTGGGCGAAATGAAC	

### Plaque formation assay

The plaque assay was also performed on DEFs, as per standard protocols^39^. After inoculation with DEV-LoxP, DEV-ΔUL54 or DEV-ΔUL54 (Revertant), the DEFs were incubated on semisolid culture medium (a 2 × MEM and methylcellulose mixture) at 37 °C for several days. The cells were then fixed with paraformaldehyde and stained with 0.5% crystal violet. Plaque areas were measured with Image-Pro Plus 6.0 (IPP 6.0), and 100 plaques were randomly chosen for each virus.

### Real-time fluorescence quantitative PCR (RT-PCR)

DEFs were infected with DEV-LoxP, DEV-ΔUL54 or DEV-ΔUL54 (Revertant). The cells were then harvested at 12, 24, 36, 48, 60, or 72 h for viral DNA replication and mRNA expression analyses. All analyses were performed independently in triplicate, and statistical significance was evaluated with the use of the unpaired Student’s t test.

To analyse DNA replication, a standard curve was first constructed. The viral DNA in the samples was then extracted according to the instructions of the Viral RNA/DNA Extraction Kit (Takara, 9766), and real-time PCR was performed with the purified nucleic acids as a template. All of the analyses were performed independently in triplicate, and statistical significance was evaluated with the use of the t test.

A nuclear run-off assay^12, 40^ and RNC extraction^41–43^ were performed on the samples before RNA isolation to investigate transcription and translation, respectively. For the nuclear run-off assay, nuclei were isolated according to the procedure of the nuclear extraction kit (Solarbio, SN0020). The infected cells were washed with PBS twice and centrifuged at 800 g for 5 min, and the cell pellet was harvested, after which the cells were resuspended in 1.0 mL of pre-cooled lysis buffer and pestled with a homogenizer for 10 sec after adding 50 μL of Reagent A. After centrifugation at 4 °C and 700 g for 5 min, the supernatant was discarded, and the sample was resuspended in 0.5 mL of pre-cooled lysis buffer. The suspension was then added to a centrifuge tube containing 0.5 mL of medium buffer and centrifuged at 4 °C and 700 g for 5 min. After the supernatant was discarded, 0.5 mL of lysis buffer was added, and the solution was centrifuged at 1000 g for 10 min. The supernatant was again discarded, and the pure nuclei were precipitated at the bottom of the tube. Finally, the nuclei were resuspended in 300 µL of runoff buffer (25 mM Tris-Cl (pH 8.0), 12.5 mM MgCl_2_, 750 mM KCl, and 1.25 mM NTP mix) and incubated at 37 °C for 15 min to complete transcription. The RNC was extracted using a common approach: infected cells were pretreated with 100 mg/mL CHX for 15 min and then washed with pre-chilled PBS. The cells were subsequently incubated in cell lysis buffer on ice for 30 min and centrifuged at 4 °C and 16200 r/min for 10 min to remove the cell debris. The supernatant was transferred to the surface of a sucrose buffer and centrifuged at 4 °C and 185000 r/min for 5 h to obtain the RNC. Total RNA, nuclear RNA, and RNC-containing RNA were isolated using RNAiso Plus (Takara, D9108A) and were reverse transcribed to cDNA (Takara, DRR047A), which served as a template for the subsequent real-time PCR. The primers used in this study are available upon request (Table 1). The relative transcription levels of the DEV UL19, UL30, UL48, gC, gD and gK genes were calculated using the 2^−ΔCt^ method.

### FISH assay

To conduct FISH, a probe was designed and synthetized by Sangon Biotech to visualize DEV UL30 mRNA. The sequence was 5′-TAGAGTCCCCAACAGATGCGAAAAGTAGTAGTCGGTG-3′, which was tagged with FITC at the 5′ terminus. DEF cells infected with DEV-derived virus were plated onto coverslips and sequentially treated with 4% paraformaldehyde, 0.2 mol/L HCl, and 100 μg/mL protease K. After prehybridization, hybridization and DAPI staining, the cells were imaged with a fluorescence microscope^44^.
